# Evaluation of Nuchal Translucency Measurement in First Trimester Pregnancy

**Published:** 2011-03-21

**Authors:** Mojgan Barati, Mahvash Zargar, Sara Masihi, Sima Taherpour

**Affiliations:** Gynecology and Obstetrics Department, Imam Khomeini Hospital, Ahvaz Jundishapur University of Medical Sciences, Ahvaz, Iran

**Keywords:** Nuchal Translucency, Chromosomal Anomaly, Karyotype

## Abstract

**Background:**

A significant number of pregnancies, particularly in women with previous histories of infertility, are associated with fetal abnormalities. Methods such as the nuchal translucency (NT) measurement enable us to identify more pregnancies with chromosomal abnormalities.

**Materials and Methods:**

This analytic cross-sectional study was performed in 446 pregnant women at 11-14 weeks gestation, from 2009 to 2010 in the Fetal Medicine Unit of Imam Khomeini Hospital, Ahvaz Jundishapur University of Medical Sciences. All NT measurements were performed by a certified sonographer using the Fetal Medicine Foundation (FMF) recommended protocol. FMF first trimester software was used for primary and secondary (adjusted) risk calculation.

**Results:**

The average maternal age was 28.5 years and 15% of mothers were ≥35 years of age. The average crown rump length (CRL), gestational age and NT thickness were 61.7, 12.4 weeks and 1.75 mm, respectively. There were 20 cases with increased adjusted risk (4.04%) and 4 cases of documented abnormal karyotype.

**Conclusion:**

In our study increased adjusted risk was 4.04%.Documented abnormal karyotype were 0.9% and 28% of total and high-risk groups who accepted amniocentesis, respectively. In this study, 50% of women with high-risk results and about half of those with abnormal karyotypes were seen in women under age 35. Knowing these risks is of utmost importance in pregnancy, particularly in patients with infertility histories.

## Introduction

A significant number of pregnancies, especially women with past histories of infertility, are associated with fetal abnormalities (1, 2). Methods such as the nuchal translucency (NT) measurement enable us to identify more pregnancies with chromosomal abnormalities such as Down syndrome (3, 4). NT is the sonographic appearance of a subcutaneous collection of fluid behind the fetal neck in the first trimester of pregnancy, which is best performed at 11-14 weeks gestation (5).

The term translucency is used irrespective of whether it is confined to the neck or envelopes the whole fetus. In fetuses with chromosomal abnormalities, cardiac defects and many genetic syndromes, the NT thickness is increased (6). Measurement of fetal NT thickness can identify a large proportion of fetuses with major defects of the heart and great arteries (7). Increased fetal NT thickness is a common phenotypic expression of fetal chromosomal defects, structural abnormalities and genetic syndromes (8).

In cases with increased NT and normal karyotype, the frequency of fetal malformations, especially heart defects, adverse pregnancy outcomes and postnatal abnormalities is related to the NT thickness (9). In the past five years large studies have shown the benefit and efficacy of NT screening for aneuploidy (10). In first trimester fetuses with no identifiable anomalies other than an isolated localized NT of 3 mm or more, 12% were shown to have an abnormal karyotype (11).

Increased NT is also associated with congenital diaphragmatic hernia (CDH) (12). Many skeletal dysplasias appear to be associated with an increased NT, which may be due to the effects of mediastinal compression or differences in collagen expression (13). Even in pregnancies with significantly increased NT and normal chromosomes, 77% of infants are born alive and healthy (12). The American College of Obstetricians and Gynecologists recommend NT, triple test and other necessary screening in all women who present for prenatal care before 20 weeks gestation (14, 15).

This study aimed to evaluate pregnant women at 11-14 weeks gestation and analyzed the proportion of high-risk and normal NT.

## Materials and Methods

**Fig 1:**
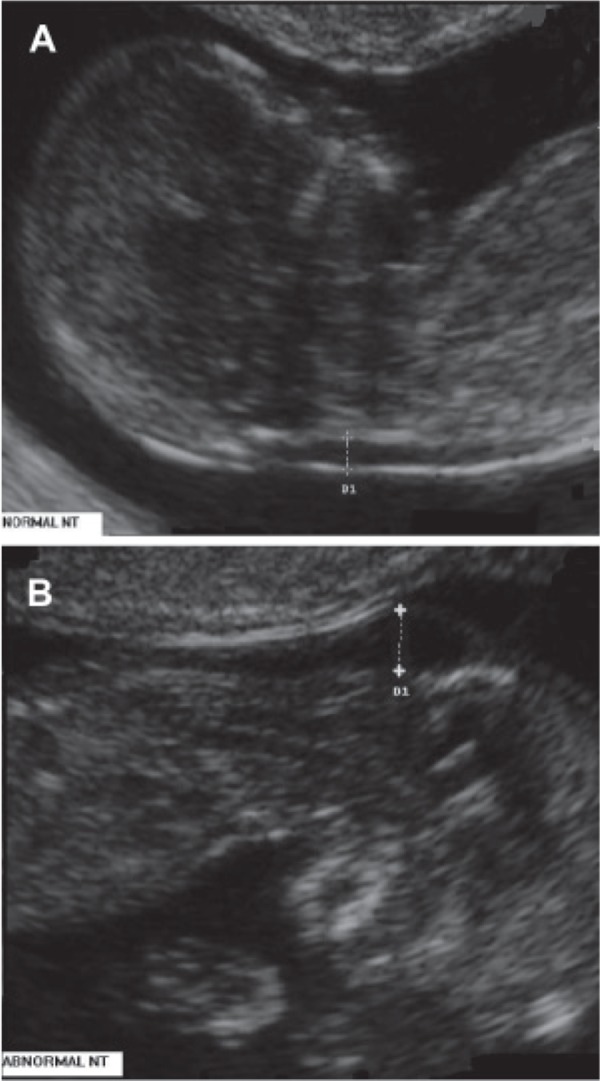
Nuchal translucency test measurements. Figure A shows a normal fetus, figure B shows ultrasound picture of a 12-week gestation fetus with trisomy 21, demonstrating increased nuchal translucency thickness.

This was an analytic cross-sectional study performed on 446 patients from the Fetal Medicine Unit in Imam Khomeini Hospital, Ahvaz Jundishapur University of Medical Sciences, Iran from 2009 to 2010. NT scans were performed at 11-14 weeks gestations with crown rump length (CRL) between 45-84 mm. A certified sonographer performed all NT scans using the Fetal Medicine Foundation (FMF) recommended protocol. The ultrasound machine was Me dison V20.First trimester affected fetuses have a subcutaneous collection of fluid behind the neck which can be easily envisioned by ultrasound as NT (Fig 1B).

The study was approved by the University Hospital and Ahwaz Jundishapur University of Medical Sciences Ethics Committees, and all subjects granted informed consent to participate. For performing NT scans, the magnification of the image should be such that the fetal head and thorax occupy the whole screen as well as a middle sagittal view of the face should be obtained. The fetus should be in a neutral position, with the head aligned with the spine. When the fetal neck is hyper-extended, the measurement can be falsely increased and when the neck is flexed, the measurement can be falsely decreased. Care must be taken to distinguish between fetal skin and amnion.

The widest part of translucency must always be measured. Measurements should be taken with the inner border of the horizontal line of the calipers placed on the line that defines the nuchal translucency thickness. The crossbar of the caliper should be such that it is hardly visible as it merges with the white line of the border, not in the nuchal fluid.

FMF first trimester software was used for primary and secondary (adjusted) risk calculation. We used only NT for FMF software risk calculations due to unavailability of a Kryptor machine for measuring pregnancy associated plasma protein-A (PAPP-A) and free beta-human chorionic gonadotropin (β-hCG) in our centre. We divided the results as low, intermediate and high-risk, and offered amniocentesis for high-risk women. Karyotype results of amniocentesis were checked.

### Statistical analysis

All analysis was done using SPSS 16. Data were presented as numbers and percentages.

## Results

The mean maternal age was 28.5 ± 6 years. Eightyfive percent of mothers were below 35 years old and the remainder (15%) were 35 years of age and older (Table 1).

**Table 1:** Age distribution in total patients


Age group	Frequency	Percentage

**<30**	299	67
**31-34.9**	76	18
**≥35**	71	15
**Total**	446	100


The lowest CRL was 45 mm and the highest was 84 mm (average: 61.7 ± 9.82). Of these, 22% of CRL ranged between 45 to 54 mm, 42.2% between 55 to 64 mm, 25.3% between 65 to 74 mm and 10.5% between 75 to 84 mm. The mean gestational age was 12.4 ± 0.67 weeks (range: 11-13.6 weeks).

The average NT thickness was 1.75 mm. Distribution of NT and age with abnormal karyotype are shown in table 4. In reviewing the relationship between CRL and NT, a significant relationship was seen, which meansthe NT thickness increased by increasing the CRL length (p ≤ 0.001). There was no significant relationship between maternal age and NT (p=0.39).

There was a significant relationship between gestational age and NT (p ≤ 0.001), that indicated with increasing gestational age, NT increased. There were 20 cases with increased adjusted risk (4.04%) and 4 cases of documented abnormal karyotype (0.9% and 28% of total and high-risk groups who accepted amniocentesis, respectively). About 50% of women with high-risk results were less than 35 years of age (Table 3), in which amniocentesis was offered for this group. Of these, 14 women accepted amniocentesis and 6 declined. Of the 14 cases which amniocentesis was performed, 4 (28%) abnormal karyotypes were observed (Table 2).

**Table 2:** Age distribution in high-risk patients


Age group	No (%)

**< 24**	1 (5)
**25-29**	4 (20)
**30-34**	5 (25)
**> 35**	10 (50)


Of 6 cases with no amniocentesis, 3 had apparently normal fetuses at birth , one case was lost to follow up, one case ended in intra uterine fetal death (IUFD) at 26 weeks and in the last case pregnancy was terminated due to fetal major thalasemia diagnosed by chorionic vilus sampling in first trimester.

## Discussion

We observed 20 cases of increased adjusted risk and 4 cases of documented abnormal karyotype, which equaled 0.9% and 28% of total and highrisk groups that accepted amniocentesis, respectively.

According to a study by Monni et al. on the records of 32000 fetuses from 11 to 14 weeks gestation, a total of 16654 fetuses were studied by both NT measurement and nasal bone evaluation.

The median maternal age was 32 years (range: 14-49). In 854 fetuses (5.1%), NT was greater than the 95th percentile and of these, 744 (87.1%) had a normal karyotype. Among 141 (0.8%) diagnosed cases of chromosomopathies, there were 96 cases of trisomy 21. (16).

Zoppi et al. (17) had reported on NT screening in 5532 fetuses from 5425 pregnancies (85 twins, 8 triplets, 2 quadruplets). The visualization of the fetal profiles was obtained in 5525 fetuses (99.8%). In 5491 fetuses (99.4%) the nasal bone was present and in 34 cases (0.6%) it was absent. Fetal karyotype and pregnancy outcome were available in 3503 pregnancies of which 40 chromosomal abnormalities were diagnosed as follows: trisomy 21 (n=27), trisomy 18 (n=5), trisomy 13 (n=2), Turner syndrome (n=3), partial trisomy 9 (n=1) and others (n=2) (17). Sepulveda et al. screened 1287 consecutive singleton pregnancies in a study conducted over a three year period. The median maternal age was 33 years (range: 14-47), with 456 (35.4%) women aged 35 years or older at the time of the scan. Overall, 110 fetuses (8.5%) had NT thickness greater than the 95th percentile for gestational age and 25 (1.9%) had an absent nasal bone. Trisomy 21 was diagnosed in 31 cases.

Among these, the NT thickness was increased in 28, and the nasal bone was absent in 13 (detection rates of 90.3% and 41.9%, respectively) (18). Cicero and co-workers reported that the fetal profile was successfully examined in 5851 (98.9%) cases.

**Table 3:** Outcome of amniocentesis and cytogenetic analysis in pregnant women


Group	Total	Amniocentesis		Culture of amniotic fluid	
		Yes	No	Normal karyotype	Abnormal karyotype

**High risk**	20	14	6	10	4
			normal at birth (n=3)		Down syndrome (n=1)
			IUFD (n=1)		Turnersyndrome (n=1)
			Major Thalassemia (n=1)		47XYY (n=1)
			Notfollowed (n=1)		47XX-mar (n=1)
**Intermediate risk**	4	-	-	-	-
**Low risk**	422	-	-	-	-


In 5223 out of 5851 cases the fetal karyotype was normal and in 628 cases it was abnormal (19).

Kagan et al. studied 11315 pregnancies. The median maternal age was 34.5 (range: 15-50) years and the median fetal crown-rump length was 64 (range 45-84) mm. The fetal karyotype was abnormal in 2168 (19.2%) pregnancies. The incidence of chromosomal defects increased with NT thickness from approximately 7% for those with NT between the 95th percentile for CRL and 3.4 mm, to 75% for NT of 8.5 mm or more. In the majority of fetuses with trisomy 21, the NT thickness was less than 4.5 mm, whereas in the majority of fetuses with trisomies 13 or 18 it was 4.5-8.4 mm, and in those with Turner's syndrome it was 8.5 mm or more (20).

## Conclusion

In our study increased adjusted risk was 4.04% and documented abnormal karyotype were 0.9% and 28% of total and high-risk groups who accepted amniocentesis, respectively. We showed that 50% of women with high-risk results and approximately half of abnormal karyotypes were noted in women under the age of 35 years. Therefore, if case screening tests are restricted to only women over the age of 35 years there is a chance that younger women with abnormal fetuses will be missed. In this study we showed that increased NT can identify not only trisomy 21, but also other numerous chromosomal anomalies such as Turner’s syndrome, 47 XYY and 47XX-mar. Knowing these risk factors may be important in pregnancy, particularly in patients with histories of infertility.

## References

[1] Allen VM, Wilson RD, Cheung A (2006). Genetics Committee of the Society of Obstetricians and Gynaecologists of Canada (SOGC), Reproductive Endocrinology Infertility Committee of the Society of Obstetricians and Gynaecologists of Canada (SOGC).Pregnancy outcomes after assisted reproductive technology. J Obstet Gynaecol Can.

[2] Imbar T, Tsafrir A, Lev-Sagie A, Hurwitz A, Laufer N, Holzer H (2006). Assisted reproduction technologies and the risk of fetal, chromosomal and genetic malformations. Harefuah.

[3] Hyett JA (2002). Increased nuchal translucency in fetuses with a normal karyotype. Prenat Diagn.

[4] Ebrashy A, El Kateb A, Momtaz M, El Sheikhah A, Aboulghar MM, Ibrahim M (2010). 13-14-week fetal anatomy scan: a 5-year prospective study. Ultrasound Obstet Gynecol.

[5] Brizot ML, Carvalho MH, Liao AW, Reis NS, Armbruster- Moraes E, Zugaib M (2001). Firsttrimester screening for chromosomal abnormalities by fetal nuchal translucency in a Brazilian population.Ultrasound Obstet Gynecol. Ultrasound Obstet Gynecol.

[6] Nicolaides KH, Wegrzyn P (2005). Fetal nuchal translucency. Ginekol Pol.

[7] Carvalho JS, Mavrides E, Shinebourne EA, Campbell S (2002). Thilaganathan B.Improving the effectiveness of routine prenatal screening for major congenital heart defects. Heart.

[8] Souka AP, Snijders RJ, Novakov A, Soares W, Nicolaides KH (1998). Defects and syndromes in chromosomally normal fetuses with increased nuchal translucency thickness at 10-14 weeks of gestation. Ultrasound Obstet Gynecol.

[9] Saldanha FA, Brizot Mde L, Moraes EA, Lopes LM, Zugaib M (2009). Increased fetal nuchal translucency thickness and normal karyotype: prenatal and postnatal follow-up. Rev Assoc Med Bras.

[10] Moore KL, Persaund TVN, Moore kl, persaund TVN (1998). The beginning of development:
The first week. The
Developing Human: Clinically Oriented Embryology.

[11] Levi CS, Lyons EA, Lindsay DJ (1988). Early diagnosis of nonviable pregnancy with endovaginal US. Radiology.

[12] Guariglia L, Rosati P (2000). Transvaginal sonographic detection of embryonic-fetal abnormalities in early pregnancy. Obstet Gynecol.

[13] Paul C, Zosmer N, Jurkovic D, Nicolaides K (2001). A case of body stalk anomaly at 10 weeks of gestation. Ultrasound Obstet Gynecol.

[14] McGee DC (2008). Evaluation of first-trimester tricuspid regurgitation for Down syndrome screening. J Perinat Neonatal Nurs.

[15] Nicolaides KH, Spencer K, Avgidou K, Faiola S, Falcon O (2005). Multicenter study of first-trimester screening for trisomy 21 in 75 821 pregnancies: results and estimation of the potential impact of individual risk-orientated two-stage first-trimester screening. Ultrasound Obstet Gynecol.

[16] Monni G, Zoppi MA, Ibba RM, Floris M, Manca F, Axiana C (2005). Nuchal translucency and nasal bone for trisomy 21 screening: single center experience. Croat Med J.

[17] Zoppi MA, Ibba RM, Axiana C, Floris M, Manca F, Monni G (2003). Absence of fetal nasal bone and aneuploidies at firsttrimester nuchal translucency screening in unselected pregnancies. Prenat Diagn.

[18] Sepulveda W, Wong AE, Dezerega V (2007). First-trimester ultrasonographic screening for trisomy 21 using fetal nuchal translucency and nasal bone. Obstet Gynecol.

[19] Cicero S, Rembouskos G, Vandecruys H, Hogg M, Nicolaides KH (2004). Likelihood ratio for trisomy 21 in fetuses with absent nasal bone at the 11-14-week scan. Ultrasound Obstet Gynecol.

[20] Kagan KO, Avgidou K, Molina FS, Gajewska K, Nicolaides KH (2006). Relation between increased fetal nuchal translucency thickness and chromosomal defects. Obstet Gynecol.

